# Arthroscopic side-to-side repair for complete radial posterior lateral meniscus root tears

**DOI:** 10.1186/s12891-020-3156-1

**Published:** 2020-02-28

**Authors:** Hongwu Zhuo, Qiang Chen, Fugui Zhu, Jian Li

**Affiliations:** Fuzhou Second Hospital Affiliated to Xiamen University, No.47, Shang Teng Street, Cang Shan District, Fuzhou, China

**Keywords:** Side-to-side, Repair, Radial, Meniscus root, Tear

## Abstract

**Background:**

The aim of this study was to determine the radiographic, second-look, and functional outcomes after arthroscopic side-to-side repair for complete radial posterior lateral meniscus root tears (PLMRTs).

**Methods:**

Patients who underwent arthroscopic side-to-side repair for complete radial PLMRTs were identified. Clinical assessment consisted of symptoms (locking, catching, giving way and effusion), examinations of joint-line tenderness and McMurray test, and subjective scores of International Knee Documentation Committee (IKDC), Lysholm, and Tegner. In addition, postoperative MRI scan and second-look arthroscopy were performed to assess the healing status of the repaired meniscus.

**Results:**

Twenty-nine patients met the inclusion criteria. The mean age was 25.41 years. The mean follow-up period was 26.68 months. During the follow-up, none of the patients had symptoms of meniscal retear, lateral joint-line tenderness or a positive McMurray test. The postoperative subjective scores of IKDC, Lysholm, and Tegner improved significantly compared to the preoperative values (*P* = 0.01). Postoperative MRI scan showed that 28/29 (96.6%) patients achieved meniscus healing. Twenty-two patients underwent second-look arthroscopy, among whom 19 (86.4%) patients showed complete meniscus healing and 3 (13.6%) patients showed partial healing.

**Conclusion:**

Arthroscopic side-to-side repair was a valuable surgical repair technique for complete radial PLMRTs, which leaded to significant improvements in both objective and subjective functional outcomes with a high rate of meniscus healing.

**Level of evidence:**

Level IV, case series.

## Background

Posterior lateral meniscus root tears (PLMRTs) are defined as tears that occur within 9 mm of the posterior lateral meniscus insertion or as avulsions of the insertion [1]. According to Ahn’s classification which is based on the arthroscopic findings, 4 types of PLMRTs are described: (1) radial tear with oblique flap, (2) longitudinal cleavage between the bony insertion and meniscal femoral ligament (MFL) insertion, (3) acute T-type, and (4) chronic inner loss type [2]. Among these, radial tear is the most common type.

Previous studies have shown that complete radial PLMRTs disrupt the circumferential fibers of the meniscus and lead to loss of hoop stress, which results in an abnormal load sharing and unacceptable peak pressure in the tibiofemoral joint [1, 3]. Thus, surgical repair should be attempted as much as possible. Currently, there are 2 common techniques of surgical repair described in the literature for complete radial PLMRTs: transtibial pull-out technique and side-to-side repair technique [4–10]. Transtibial pull-out technique has been reported to successfully restore the meniscal functions by securing the meniscus to its original anatomic insertion site on the tibia [4, 5]. However, when the root fragment of the meniscus is large enough, side-to-side repair technique is theoretically biomechanically superior as it repairs the meniscus anatomically without changing its native physiologic properties [6–10].

To our best knowledge, there is still limited data in the literation regarding the clinical outcomes after arthroscopic side-to-side repair for complete radial PLMRTs. Therefore, the aim of this present study was to assess the radiographic, second-look, and functional outcomes after arthroscopic side-to-side repair for complete radial PLMRTs. We hypothesized that this surgical procedure would result in significant improvements in both objective and subjective functional outcomes with a high rate of meniscus healing.

## Methods

**Study design**

From March 2014 to March 2017, the medical records of patients who underwent arthroscopic surgery for PLMRTs at our institution were identified. The inclusion criteria were (1) patients who were diagnosed with complete radial PLMRTs and underwent arthroscopic side-to-side repair, and (2) patients with a minimum 2-year follow-up. The exclusion criteria were (1) age older than 60 years, (2) significant osteoarthritis of the joint (Kellgren-Lawrence grade III or IV), or (3) previous surgery of the same knee. As complete radial PLMRTs occurred frequently associated with anterior cruciate ligament (ACL) injury, so concomitant ACL injury was not an exclusion criterion for this study.

**Fig. 1:**
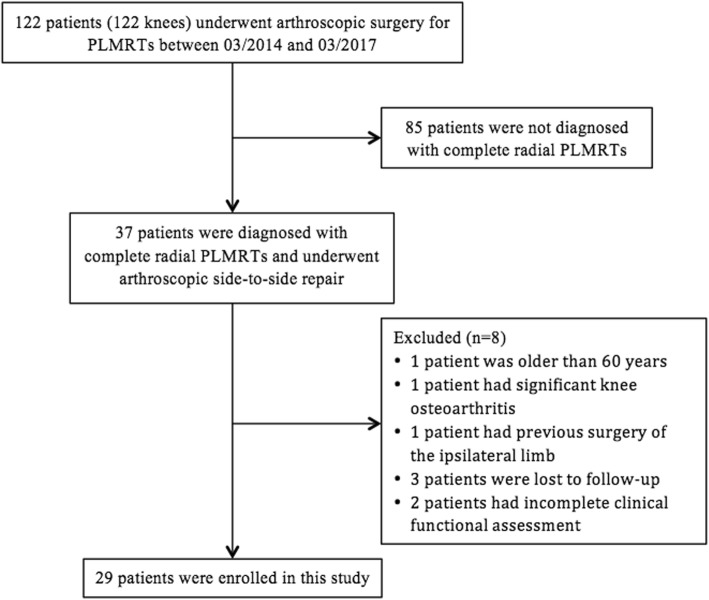
Flow chart of this study

A total of 122 patients (122 knees) who underwent arthroscopic surgery for PLMRTs at our institution were identified (Fig. 1). Of these, 37 patients were diagnosed with complete radial PLMRTs and all these patients underwent arthroscopic side-to-side repair. Among them, one patient was older than 60 years, one patient had significant knee osteoarthritis, one patient had previous surgery for tibial plateau fracture, three patients were lost to follow-up with a follow-up period less than 2 years, and two patients had incomplete clinical functional assessments. Therefor, we studied the remaining 29 patients (29 knees).

This study received approval from our institutional review board. All patients provided signed informed consent to allow their clinical and radiological data to be used for research programs.

### Surgical technique

**Fig. 2:**
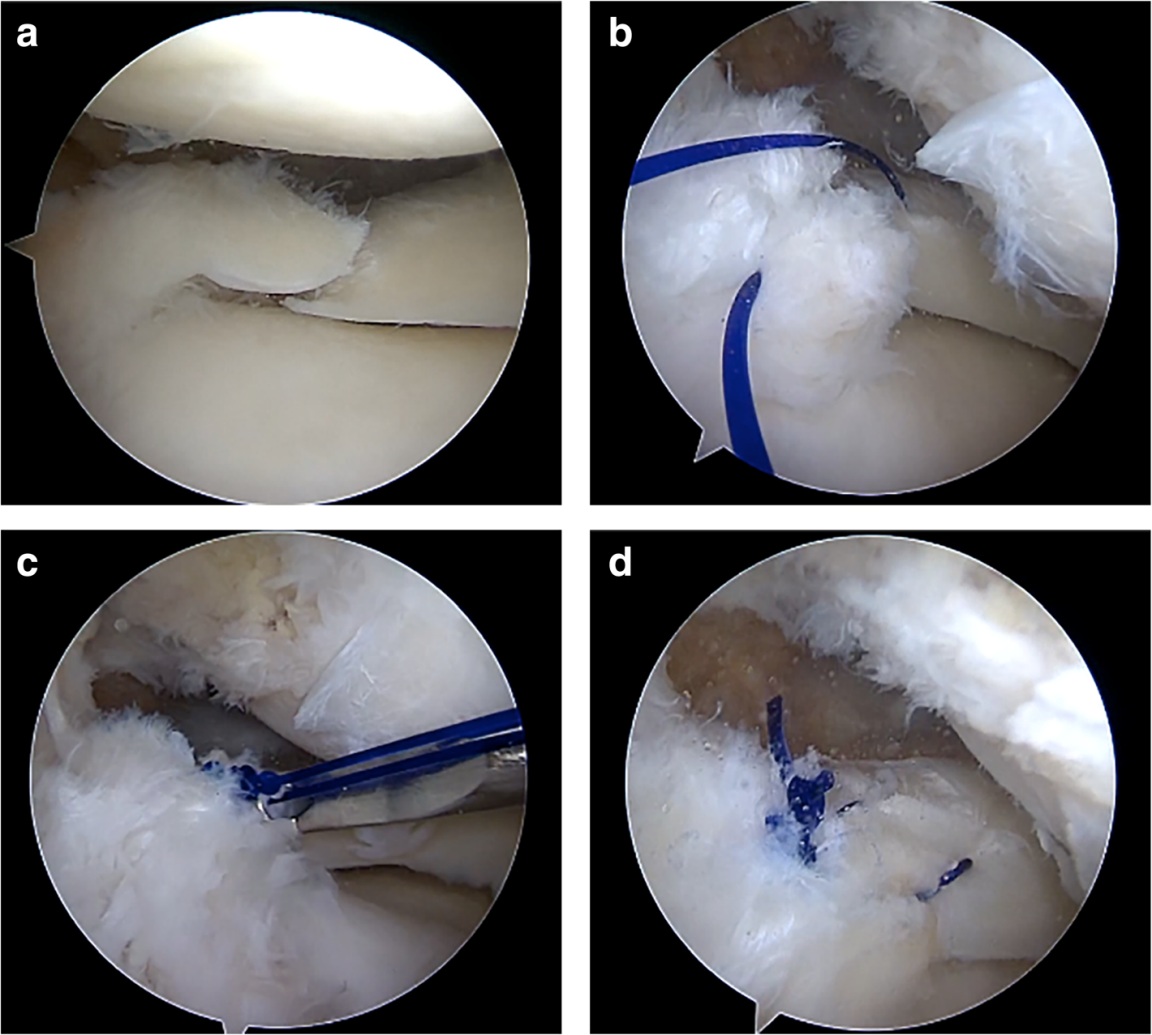
Arthroscopic side-to-side repair for complete radial PLMRTs (left knee). **a** A complete radial PLMRT; **b** A PDS suture was passed through both sides of the torn meniscus; **c** The two ends of the PDS suture were tied using a knot pusher; **d** A total of 2 stitches were required to create a stable repair

All surgeries were performed under general anesthesia. The patients were placed in a supine position. An arthroscopic evaluation was performed using two standard anterior knee arthroscopy portals. If the ACL was torn, the lateral meniscus was addressed before ligament reconstruction. The knee was placed in the figure-of-4 position. The torn edge of the meniscus was refreshed with a motorized shaver (Fig. 2a). A suture hook loaded with a No. 2 polydioxanone (PDS; Ethicon, Somerville, NJ) suture was introduced through the anterolateral portal and pierced the outer part of the meniscus downward approximately 5 mm away from the torn edge. Then, a suture hook loaded with a lasso loop was introduced through the anteromedial portal and pierced the inner part of the meniscus downward to bring the inferior end of the PDS suture through the inner part of the meniscus upward (Fig. 2b). Next, both of the two free ends of the PDS suture were retrieved and tied with four to five simple knots using the arthroscopic knot pusher (Fig. 2c).

The same procedure was repeated and it required usually 2 or 3 stitches to form a firm repair (Fig. 2d).

### Postoperative rehabilitation

Postoperatively, the leg was immobilized with a knee brace in full extension. Quadricep-strengthening exercises were started on the second day. Passive knee flexion was started at 2 weeks postoperatively, while active knee flexion was started at 4 weeks postoperatively. Partial weight bearing was allowed at 6 weeks postoperatively and full weight bearing was allowed at 8 weeks postoperatively. Normal activities were permitted at 6 months postoperatively.

### Assessment

Demographic data were collected from the database, including age, sex, side of injury, time from injury to surgery, and concomitant surgical procedures. The patients were assessed preoperatively; at 1, 3, 12, and 24 months postoperatively; and at the final follow-up.

The functional outcomes were assessed by both objective and subjective measures. These measures included (1) the symptoms of meniscus retear, such as locking, catching, giving way and effusion; (2) examinations of joint-line tenderness and McMurray test; and (3) International Knee Documentation Committee (IKDC) score, Lysholm score, and Tegner score. According to Barrett’s criteria, a repaired meniscus was considered clinical healing if there was no joint line tenderness, no effusion and a negative McMurray’s test [11].

At the final follow-up, MRI scan (3.0-T MR System, Signa Excite, GE Medical Systems, Waukesha, Wisconsin, USA) was obtained for assessment of the healing status of the repaired meniscus. A repaired meniscus was considered failure of healing if one of following MRI signs was present: (1) “ghost sign”, the absence of an identifiable meniscus on the sagittal sequence or high signal replacing the normal dark meniscus [12]; (2) meniscal extrusion, a meniscal sagittal displacement of > 3 mm at the level of the medial collateral ligament [13]; and (3) abnormal hyperintensity signal, which extended to the articular surface of the meniscus root [14].

For the patients who wanted to remove the implant for ACL reconstruction and agreed to undergo second-look arthroscopy, the healing status of the repaired meniscus was assessed using an arthroscopic probe and classified according to Horibe’s criteria [15]. If there was no visible surface defect with complete synovial coverage, it was defined as complete healing. If there was a small defect with synovial coverage over more than half of the torn area, it was defined as partial healing. If there was a large defect with synovial coverage over less than half of the torn area, it was defined as failure of healing.

### Statistical analysis

All statistical analyses were performed using SPSS software (IBM-SPSS statistics 22.0; New York, USA). Continuous variables were presented as the mean and standard deviation. A paired-samples t test was used to determine the differences between preoperative and postoperative quantitative variables. The significance level was set at 0.05.

## Results

### Demographic data

A total of 29 patients met the inclusion criteria and were enrolled in this study. There were 21 males and 8 females with a mean age of 25.41 ± 6.61 years (range, 17–39 years). The mean follow-up period was 26.68 ± 2.91 months (range, 24–36 months). The demographic data of the patients are summarized in Table 1.

**Table 1:** Demographic data of the patients

Variable	Value
Patients, n	29
Age, years	25.41 ± 6.61
Sex, male/female	21/8
Side, right/left	16/13
Time from injury to surgery, months	3.38 ± 4.17
Concomitant surgical procedures, n	
Anterior cruciate ligament reconstruction	26
Partial or total meniscectomy of medial meniscus	14
Debridement for cartilage damage	3
Follow-up period, months	26.68 ± 2.91

The data are shown as mean ± standard deviation or n

### Functional outcomes

During the follow-up, none of the patients complained of locking, catching, giving way or effusion. None of the patients had lateral joint-line tenderness or a positive McMurray test. Thus, the repaired meniscus was considered having a 100% clinical success rate. At the final follow-up, the subjective scores of IKDC, Lysholm, and Tegner improved significantly compared to the preoperative values (Table 2).

**Table 2:** Comparison of the preoperative and postoperative functional outcomes

Variables	Preoperative	1 month postoperatively	3 month postoperatively	12 month postoperatively	24 month postoperatively	Final follow-up	*P* Value (preoperative vs final follow-up)
IKDC score	53.4 ± 5.32	26.4 ± 2.32	47.9 ± 3.74	84.0 ± 3.09	91.4 ± 2.72	92.1 ± 2.64	.01
Lysholm score	56.3 ± 4.59	49.2 ± 4.44	88.0 ± 2.10	94.9 ± 2.59	95.1 ± 2.85	95.1 ± 2.85	.01
Tegner score	2.41 ± 0.56	0.17 ± 0.38	2.46 ± 0.67	5.27 ± 0.75	5.41 ± 0.66	5.55 ± 0.63	.01

The data are shown as mean ± standard deviation

### Radiographic and second-look outcomes

**Fig. 3:**
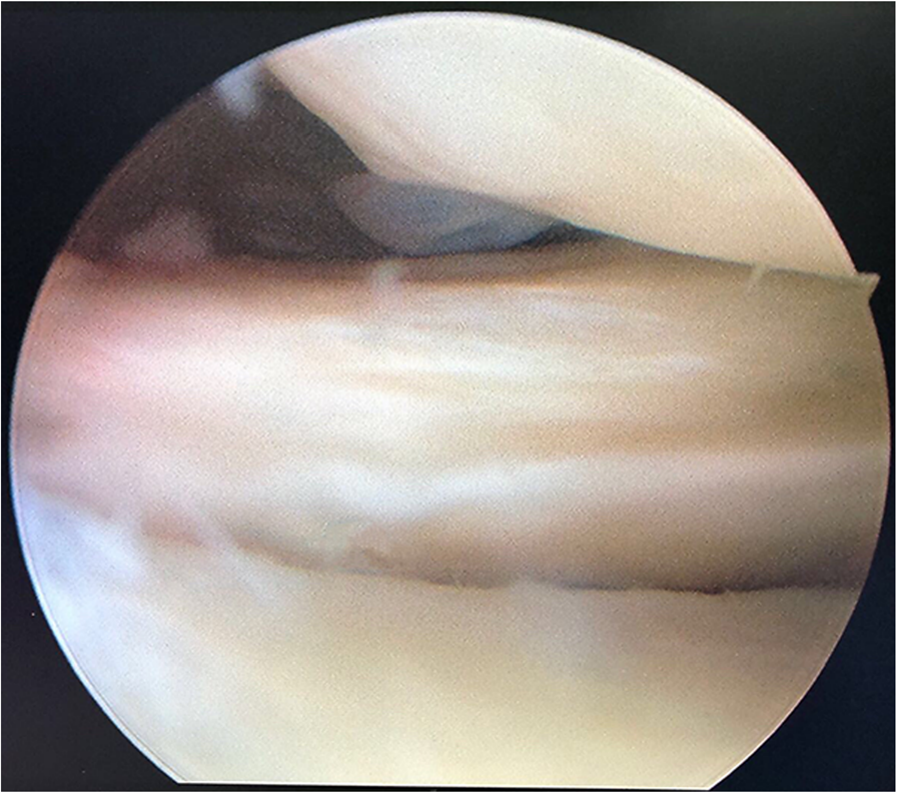
Complete meniscal healing on the second-look arthroscopy

The postoperative MRI showed that 28 (96.6%) patients achieved complete healing and 1 patient achieved failure of healing with an abnormal hyperintensity signal in the repair site. Twenty-two patients underwent second-look arthroscopy at mean 13.0 ± 1.31 months (range, 11–16 months) postoperatively. On the second-look arthroscopy, 19 (86.4%) patients showed complete meniscus healing (Fig. 3) and 3 (13.6%) patients showed partial healing. No failure of healing was detected among these 22 patients.

## Discussion

The most important finding in this present study was that the subjective scores of IKDC, Lysholm, and Tegner after arthroscopic side-to-side repair for complete radial PLMRTs got significant improvement with a clinical success rate of 100% at a mean follow-up of 26.68 months. Moreover, 96.6% of patients showed meniscus healing on the postoperative MRI. Twenty-two patients underwent second-look arthroscopy and 19 patients showed complete meniscus healing.

### Biomechanical consequences of complete radial PLMRTs

The primary biomechanical function of meniscus is to convert the tibiofemoral axial load into hoop stress during both knee extension and deep flexion [16]. Complete radial PLMRTs disrupt the continuity of the circumferential fibers and lead to loss of hoop stress, causing overload of the lateral compartment and early degenerative changes [1, 3]. A recent biomechanical study by Schillhammer showed that PLMRTs resulted in a significantly increased peak contact pressure (49%) and decreased tibiofemoral contact area (33%) [17]. The posterior lateral meniscus root is also an important secondary restraint to the rotational laxity of the knee joint. To date, several studies have shown that PLMRTs further destabilize the rotational stability of the ACL-deficient knee under a simulated pivot-shift loading [18, 19]. Overall, complete radial PLMRTs have severe biomechanical consequences and are functionally equivalent to a total meniscectomy.

### Surgical repair of complete radial PLMRTs

Arthroscopic transtibial pull-out technique and side-to-side repair technique are the 2 most common techniques of surgical repair for complete radial PLMRTs [4–10]. The former involves a bone tunnel running from the anteromedial aspect of the tibia to the anatomic attachment site of the lateral meniscal root on the tibia. Sutures are then passed through the meniscal root and through the tunnel and secured over the anterior tibia. However, the theoretical disadvantages of this technique include (1) need for a bone tunnel which might interfere with concomitant ligament reconstruction, (2) need for distal fixation which places the sutures at risk for failure, and (3) if the root fragment is large, that the outer part of the meniscus is secured to the original insertion site will cause an increased meniscal tension [20]. In contrast, side-to-side repair belongs to anatomic repair that does not change the native anatomic and physiologic properties of the meniscus.

Recently, posterior lateral meniscus root fixation using suture anchors has also been described [21]. The main concerns about this technique include anchor pullout with subsequent failure of fixation, and technical difficulty [22].

### Clinical outcomes after arthroscopic side-to-side repair for complete radial PLMRTs

To date, the studies regarding the clinical outcomes after arthroscopic side-to-side repair for treatment of complete radial PLMRTs remain limited. Ahn reported a cohort of 27 patients who underwent arthroscopic side-to-side repair for complete radial PLMRTs [6]. The clinical healing rate of the repaired meniscus was up to 100% at 1 year postoperatively. Eight of the nine patients who underwent second-look arthroscopy showed complete meniscus healing. Anderson retrospectively reviewed 8 patients who underwent arthroscopic side-to-side repair for complete radial PLMRTs [7]. The mean IKDC, Lysholm, and Tegner scores improved significantly at a mean follow-up of 70.5 months. In another study, Song analyzed a small sample of 15 patients with complete radial PLMRTs who underwent arthroscopic side-to-side repair using a Fast-Fix device [8]. At a mean follow-up of 24 months, all the patients showed clinical healing, and 86.6% of the patients showed meniscus healing on the second-look arthroscopy. Similar to these studies, this present study also showed that arthroscopic side-to-side repair could significantly improve both objective and subjective functional outcomes with a high rate of meniscus healing in patients with complete radial PLMRTs.

This study has several limitations. First, this study was a retrospective study with all the inherent limitations of a retrospective study. Second, this study included a limited number of patients. Third, the period of the follow-up was 2 years but still relatively short, and longer-term evaluations are required to evaluate long-term clinical outcomes. Fourth, this study did not involve any control group.

## Conclusions

Arthroscopic side-to-side repair was a valuable surgical repair technique for complete radial PLMRTs. This surgical procedure leaded to significant improvements in both objective and subjective functional outcomes with a high rate of meniscus healing.

## Data Availability

The raw data are available from the corresponding author upon reasonable request.

## Abbreviations

ACL: Anterior cruciate ligament
IKDC: International Knee Documentation Committee
MFL: Meniscal femoral ligament
PLMRTs: Posterior lateral meniscus root tears
